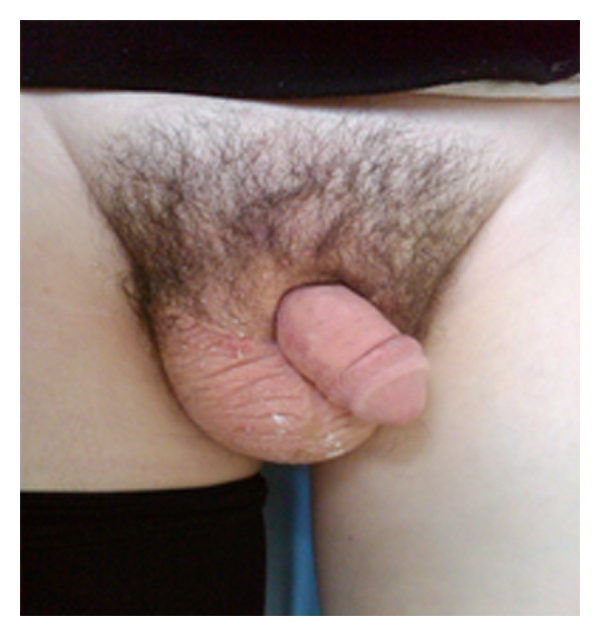# Penile Reconstruction for a Case of Genital Lymphoedema Secondary to Proteus Syndrome

**DOI:** 10.5402/2011/431536

**Published:** 2011-03-30

**Authors:** F. Ashouri, J. Manners, R. Rees

**Affiliations:** Department of Urology, RHCH, SO22 5DE Winchester, UK

## Abstract

To our knowledge penile lymphoedema secondary to Proteus syndrome has not previously been reported. Hence we report a case of a 16-year-old male who was referred with features of right hemi-hypertrophy and severe lymphoedema affecting his scrotum and penis. He had previously undergone scrotal reduction surgery at the age of 13, but had since developed worsening penile oedema. His main concern was that of cosmetic appearance prior to sexual debut, and he also complained of erectile dysfunction. An MRI confirmed gross oedema of the penile skin, but normal underlying cavernosal structure, and no other anatomical abnormality. Under general anaesthesia, the entire diseased penile skin was excised. Two full thickness skin grafts were harvested from the axillae, and grafted onto the dorsal and ventral penile shaft respectively. A compressive dressing and urinary catheter was applied for 7 days. Follow-up at 4 months confirmed complete graft take with minimal scarring, and the patient was very satisfied with the cosmetic outcome. He had also noticed a recovery in erectile activity, and feels psychologically and physically more prepared for sexual relations.

## 1. Case Presentation

A 16-year-old male was referred with features of right hemi-hypertrophy and severe lymphoedema affecting his scrotum and penis. He was the product of an uncomplicated pregnancy and delivery. The parents are of Caucasian ethnic origin and nonconsanguineous.

He had previously undergone scrotal reduction surgery at the age of 13, but had since developed worsening penile oedema. His main concern was that of cosmetic appearance prior to sexual debut, and he also complained of erectile dysfunction. He did not perceive the penis becoming hard or erect, but was able to achieve orgasm and ejaculate.

**Figure 1:**
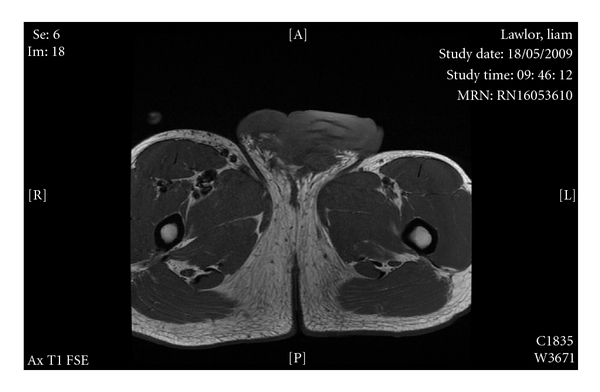


**Figure 2:**
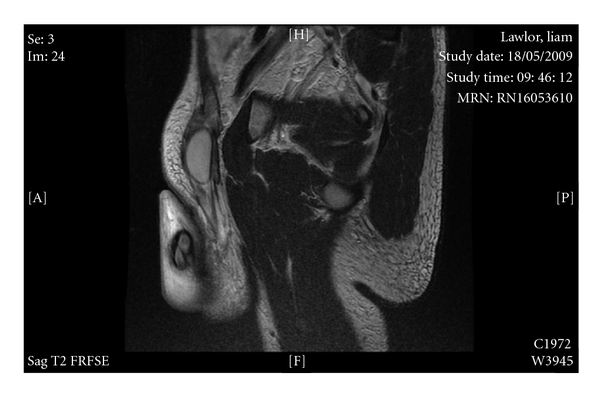


On examination, he had gross lymphoedema of the right leg, minor residual scrotal, and inguinal swelling, but severe chronic penile lymphoedema. An MRI confirmed gross oedema of the penile skin, but normal underlying cavernosal structure, and no other anatomical abnormality (see Figures 1 and 2). 

**Figure 3:**
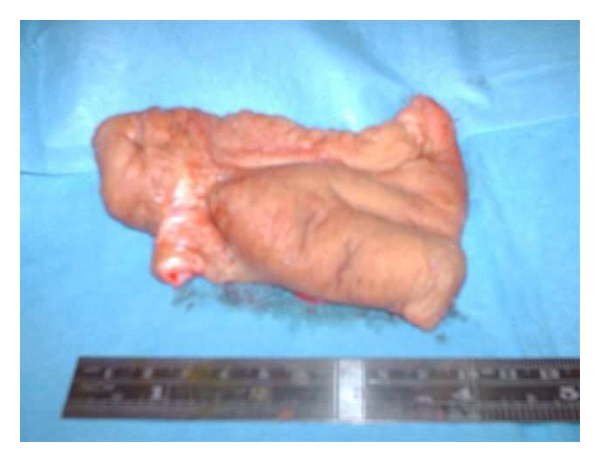


**Figure 4:**
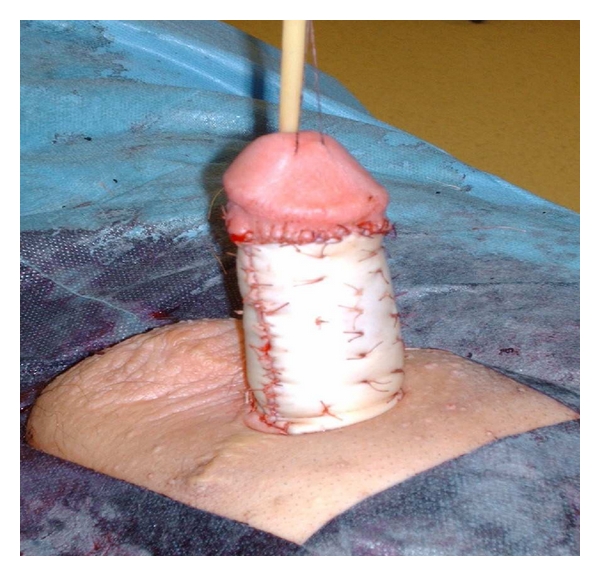


Under general anaesthesia, the entire diseased penile skin was excised. (see Figure 3). Two full thickness skin grafts (4.5 cm × 8.5 cm) were harvested from the axillae, and grafted onto the dorsal and ventral penile shaft, respectively (see Figure 4). A compressive dressing and urinary catheter was applied for 7 days.

Followup at 4 months confirmed complete graft take with minimal scarring, and the patient was very satisfised with the cosmetic outcome. He had also noticed a recovery in erectile activity, and feels psychologically and physically more prepared for sexual relations (see Figure 5).

## 2. Discussion

To our knowledge, penile lymphoedema secondary to Proteus syndrome has not previously been reported. A case by Clark et al. in 1987 reported on a 2-year-old boy with Proteus syndrome, macro-orchidism, and penile hypertrophy without any other signs of virilisation [1]. 

Proteus syndrome is a hamartomatous disorder characterised by multiple focal overgrowths that can involve any structure of the body [2]. 

It was named after the Greek god of the sea, Proteus, who was capable of assuming many forms to escape capture [3]. It is a rare genetic disorder with less than 200 cases recorded. The male: female ratio is 1.9: 1 (*n* = 96). One aetiological hypothesis is somatic mosaicism, the presence of genetically distinct populations of somatic cells, that can be lethal in the nonmosaic state [4]. It was first described by Cohen and Hayden in 1979 as a disorder characterized by the overgrowth of various tissues, hyperostoses, epidermal nevi, and connective tissue nevi [5], where overgrowth is defined as asymmetry that is greater than normal [6].

Proteus syndrome should always be considered in the differential diagnosis of genital lymphoedema. Surgical removal of the diseased skin and replacement with skin grafts is an effective means of restoring cosmetic appearance and functionality.

## Figures and Tables

**Figure 5:**